# Spiders

**Published:** 1887-07

**Authors:** 


### SPIDERS.

The spider has an interesting history, being the subject of many a curious legend and quaint superstition.

"If you wish to live and thrive,
Let the spider run alive,"

is an old-time proverb, ill-luck being supposed to overtake those who kill or even molest it. The regard which is paid by many to the life of the spider is in all probability due to the influence of an old legend which tells how, when Christ lay in the manger at Bethlehem, the spider came and spun a beautiful web over the spot where He was, thus preserving His life by screening Him from all the dangers that surrounded Him. Stories of a similar character are told about several notable persons during times of persecution. Thus there is a Hebrew tradition relating to King David. When escaping from Saul in the desert of Ziph, a spider is said to have spun a web over the cave in which he was concealed, causing his pursuers to believe that no one could be hidden there. Mohammed, too, in his flight from Mecca, is reported to have been saved by a spider and a pigeon. When his enemies reached the cave where he rested, and found a spider's web across the mouth, and a pigeon in her nest just above, they at once concluded that the place had not recently been disturbed, or the web would have been destroyed. It was a notion formerly prevalent in many parts of Scotland that should a servant willingly kill a spider, she would certainly break a piece of crockery or glass in the course of the day.

In some parts of England a species of spider is known as the "money-spinner," and prognosticates good luck. In order to propitiate it, the peasant oftentimes throws it over his left shoulder. It is said that if the

"money-spider" be found on a person's clothes, it is an omen that he will shortly have money, in allusion to which old Fuller quaintly moralizes: "When a spider is found upon our clothes, we used to say some money is coming toward us. The moral is this: Such who imitate the industry of that contemptible creature, by God's blessing weave themselves into wealth and secure a plentiful estate."

The spider has from time immemorial been a favorite charm in the cure of certain diseases. Thus in Norfolk, England, the parents of a child afflicted with whooping-cough are recommended to search for a spider in their own house, and on finding one, to hold it over the head of the child, repeating three times the following rhyme:

"Spider, as you waste away,
Whooping-cough no longer stay."

The spider must then be hung up in a bag over the mantle-shelf, and as it gradually dries up, so too, the cough will disappear. As a remedy for ague, the spider has been considered most efficacious. Some years ago, a lady in the south of Ireland was celebrated far and near for her cure of this disorder. Her remedy was a large house-spider, taken alive, enveloped in molasses or preserve. Of course, the parties were carefully kept in ignorance of what the wonderful remedy was. Sometimes a spider is put into a goose-quill, well sealed and secured, and hung about the patient's neck. Dr. Graham, in his *Domestic Medicine*, prescribed spider's webs for ague and intermittent fever; and also names powder made of spiders given for the ague, and mentions his knowledge of a spider having been sewn up in a bag and worn as periapt round the neck to charm away the ague. Another remedy consists in inclosing the spider between two halves of a nutshell, and wearing it round the neck.

The spider is occasionally used in Sussex in cases of jaundice; many an old doctress prescribed "a live spider rolled up in butter." Once more, an old writer, speaking of warts, has the following quaint remarks; "Some chirurgeons there be that cure warts in this manner: they take a spider's web, rolling the same up on a round heap like a ball, and laying it upon the wart, they set fire on it, and so burn it to ashes, and by this way and order the warts are eradicated, that they never after grow again."—*Our Record.*

---

*Problems of Nature* is the name of a Chicago weekly publication, chiefly devoted to the promulgation of a theory of natural causes and effects, at variance with all recognized systems of philosophy. It also has a miscellaneous advertising department; is sent by mail to regular subscribers, and is now well on towards its third yearly volume. It would seem that these facts would entitle it to rate, under the post office regulations, as second-class matter, but a recent decision of the Postmaster General. refuses to accord to it the privilege of this rating.

Without having seen the grounds of this decision, it would seem that if the rule were to be generally applied it would amount to a meddlesome supervision of the "press" and an invidious proscription of distasteful newspaper publications.

